# A maximum-entropy model for predicting chromatin contacts

**DOI:** 10.1371/journal.pcbi.1005956

**Published:** 2018-02-05

**Authors:** Pau Farré, Eldon Emberly

**Affiliations:** Department of Physics, Simon Fraser University, Burnaby, BC V5A1S6, Canada; Rutgers University, UNITED STATES

## Abstract

The packaging of DNA inside a nucleus shows complex structure stabilized by a host of DNA-bound factors. Both the distribution of these factors and the contacts between different genomic locations of the DNA can now be measured on a genome-wide scale. This has advanced the development of models aimed at predicting the conformation of DNA given only the locations of bound factors—the chromatin folding problem. Here we present a maximum-entropy model that is able to predict a contact map representation of structure given a sequence of bound factors. Non-local effects due to the sequence neighborhood around contacting sites are found to be important for making accurate predictions. Lastly, we show that the model can be used to infer a sequence of bound factors given only a measurement of structure. This opens up the possibility for efficiently predicting sequence regions that may play a role in generating cell-type specific structural differences.

## Author summary

The three-dimensional folding of DNA inside the nucleus into specific conformations is necessary for the proper functioning of cells. These structures can be measured by chromosome conformation capture methods (Hi-C) that report the number of times that each pair of genomic sites are found in proximal location in a cell population experiment. A number of protein complexes that bind to the DNA have been discovered to be responsible for the stabilization of such conformations. However, identifying the precise relation between the positioning of binding proteins and the resulting structures is still an open problem. Here we present a maximum-entropy method able to predict Hi-C contact probabilities from a sequence of binding factors without the need of performing any polymer simulations. We envision that this method will allow experimentalists to efficiently calculate the expected structural effect of altering the sequence of binding factors. In addition, we also show that our model is capable of solving the inverse problem, namely predicting the underlying sequence of binding factors from a set of observed contact probabilities.

## Introduction

In higher-complexity organisms, the packaged molecule of DNA inside the nucleus of a cell consists not only of the DNA polymer but also a large number of DNA-associated protein and RNA complexes that together form what is known as chromatin. These complexes bind to the DNA by either sequence affinity or by interactions with other bound factors [1], and their presence is known to be correlated with the 3D conformation of the DNA polymer [2]. They impact the structure of the DNA on many length scales: from the packing of nucleosomes at the smallest scale (∼200 basepair) to the stabilization of loops and genomic domains on much larger scales (∼10-100 kilobasepair). As a result, they regulate a host of important cell functions from DNA replication to gene expression [3, 4]. Developing models that can predict how chromatin folds given only the locations of bound factors is of key importance to better understand how they regulate such processes by shaping DNA structure.

The average structure of DNA over a population of cells can be measured genome-wide using a high-throughput DNA sequencing method known as Hi-C [5]. Briefly, contacting sites in the genome are cross linked; then the DNA is fragmented and the contacting pairs are sequenced. These sequenced pairs are then used to construct a contact map at a given spatial resolution that gives the number of times any pair of sites along the genome were found to be in contact. Analysis of the contact maps have shown that chromatin can be classified into structural types such as A/B compartments [5, 6] or topologically associated domains (TADs) [7] just from the spatial distribution of contacts.

These structural features are known to be strongly correlated with different types of DNA-bound factors. The locations of these bound factors along the genome constitute a form of sequence that helps drive the folding of the DNA—similar to the specific sequence of amino acids that drives a protein to fold. High throughput methods can provide the binding locations of such factors on a genome-wide scale [8]. Interestingly, despite tens to hundreds of different chromatin associated factors, clustering of their binding locations shows that there are only few unique bound states [9–13] (similar to the grouping of amino acids into just hydrophobic and polar types). With the richness of this structural and sequence data for chromatin, predictive models that aim to solve the chromatin folding problem—namely predicting the structure of DNA inside a cell given only the locations of the DNA-bound chromatin factors—are now being developed.

Recent modeling efforts have used polymer-based models whose parameters can be tuned to reproduce experimental observations, such as the contact map from Hi-C. One set of approaches tries to find the best 3D polymer structure that is consistent with the constraints imposed by the Hi-C contact map [14–19]. Other methods include bound factors by adding sequence-specific interactions to a given polymer model for the DNA [20–33]. These approaches have been successful in showing how interactions between factors together with topological constraints may be responsible for the observed chromatin structures. Challenges involve continuing to improve the physics of the interacting polymer model using data-driven methods and the time-consuming process of carrying out the polymer simulations.

Complementing the polymer simulation methods are purely statistical approaches that aim to predict a contact map instead of a full 3D structure. Sexton et al. [34] developed a statistical model based on site-specific scaling factors that could predict a matrix of expected counts. Other work included sequence information by fitting a pairwise interaction model for the DNA-bound factors that could then predict the probability of contact between pairs of sites given only the sequence at those locations [35]. However it did not include an important effect that is present in polymer simulations, namely the role of neighbors.

A growing body of experimental evidence supports the importance of the local sequence neighborhood of bound factors in mediating contacts between pairs of genomic sites [36]. In particular the probability of contact between two sites *i* and *j* at a certain genomic distance apart is altered if some site *k* in the neighborhood of *j* is attracted to *i*. Sites *k* and *i* will spend a fraction of the time in contact, thus altering the effective polymer distance between *i* and *j* (see S1 Fig). Here, our aim is to take such effects into account and predict the probability of two sites being in contact (i.e. the contact map) given a local sequence neighborhood. The results presented in the following sections demonstrate how these probabilities can be modelled by a maximum entropy distribution. In addition, by formulating the problem in a Bayesian fashion, we are not only able predict probabilities of contact from an experimentally measured sequence of bound factors, but also predict the probability of a site having a particular bound factor using only the measured local Hi-C contact map

## Results

### Maximum entropy model

**Fig 1:**
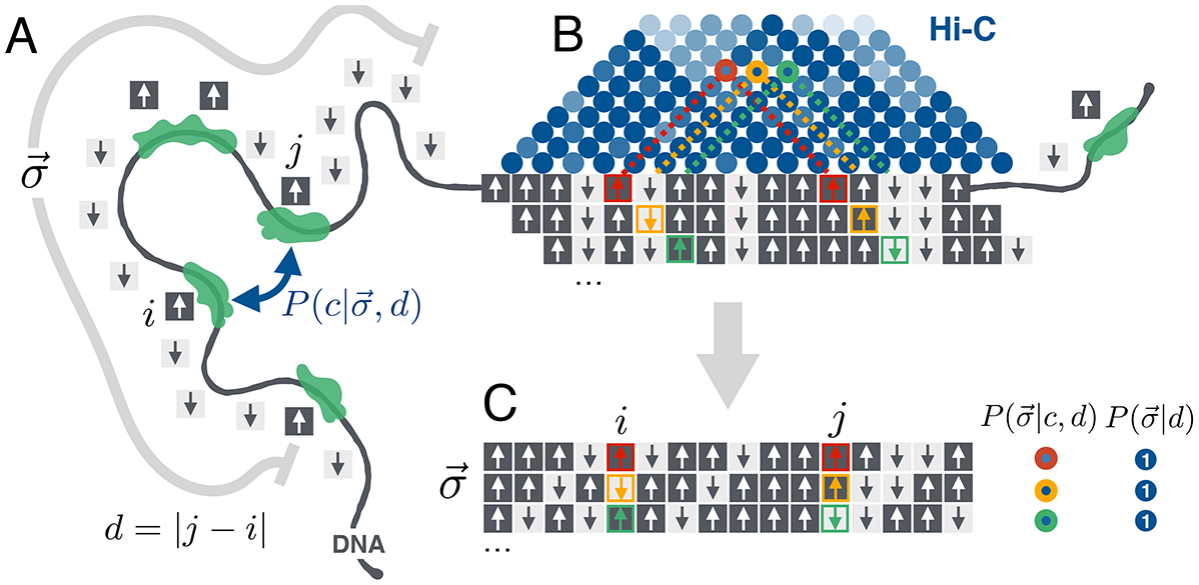
Schematic of model for predicting chromatin contacts from sequence. (A) The DNA was discretized into sites of finite size and the bound-factor sequence at each site was categorized into one of two bound states (spin-up or spin-down). The probability of contact, $P(c|\vec{\sigma },d)$, between two sites separated by a genomic distance, *d* = |*j* − *i*|, depends on the sequence neighbourhood $\vec{\sigma }$ situated around them (shown as gray line). (B) Hi-C data measures the number of times pairs of sites were found in contact (shown as heat map above). For each pair of sites *i* and *j* at a given genomic distance *d* = |*j* − *i*|, one can extract a local sequence neighborhood $\vec{\sigma }$ around the sites. (C) Schematic of data used for fitting the maximum-entropy model. The collection of all sequence neighborhoods at a given genomic distance *d* was extracted by scrolling along the genome. The statistical weights associated to each neighborhood were given by either the number of times they were observed in contact from Hi-C (left column), or occurrence in the genome (right column). These weights were implemented in the calculation of the statistical averages used as constraints on the maximum entropy distributions, $P(\vec{\sigma }|c,d)$ and $P(\vec{\sigma }|d)$ respectively.

Fig 1 shows a schematic of our model and resulting calculation. First, the genome is discretized into non-overlaping sites of fixed size. A particular chromatin state *σ*_*k*_ is assigned to to each site *k* based on the bound chromatin factors there. As mentioned in the introduction, only a few bound states exist and for simplicity we classify each site into only one of two possible sequence states corresponding to active/euchromatic and inactive/heterochromatic DNA, respectively labeled as spin-up (*σ*_*k*_ = 1) and spin-down (*σ*_*k*_ = −1) analogous to the physics of ferromagnets (see Fig 1(A) and Materials and methods for details).

With respect to structure, Fig 1(A) and 1(B) shows two sites *i* and *j* separated by a genomic distance, *d* = |*j* − *i*|, that have a certain chance of forming a contact that we assume is determined by the sequence neighborhood, $\vec{\sigma }$, situated around them. Hi-C data gives an estimate of this chance by reporting the number of times *n*_*ij*_ that this pair formed contacts, and hence the likelihood that their sequence neighborhood will form a contact (Fig 1(B)). We denote this probability of contact as $P(c|\vec{\sigma },d)$ which depends on the genomic distance *d* between sites and sequence neighborhood $\vec{\sigma }$ (here *c* indicates that the two sites are in contact; *c* is therefore one of the values of the random variable *z*: *z* = *c* if contact and $z=\bar{c}$ otherwise). The neighborhood $\vec{\sigma }$ is a vector containing *N* sites built from the union of sequence windows situated around the contacting sites *i* and *j* and thus depends on *d* (see Fig 1(B) and 1(C), Materials and methods and S2 Fig for further details).

Using Bayes’ rule we can rewrite $P(c|\vec{\sigma },d)$ as

$$\begin{array}{c} P\left(c|\vec{\sigma },d\right)=\frac{P\left(\vec{\sigma }|c,d\right)P\left(c|d\right)}{P(\vec{\sigma }|d)}, \end{array}$$ (1)

where $P(\vec{\sigma }|c,d)$ and $P(\vec{\sigma }|d)$ are the probability of observing the given sequence neighborhood $\vec{\sigma }$ given that it has formed a contact and regardless of contact, respectively. *P*(*c*|*d*) is the sequence-independent probability of contact at a separation *d* and can be estimated directly from the Hi-C experiments. In principle, Hi-C data could also be used to directly estimate $P(\vec{\sigma }|c,d)$ since the contact map gives the number of times that a given neighborhood was found to form a contact (see Fig 1C). Similarly, $P(\vec{\sigma }|d)$ could be estimated from the frequency of occurrence of that sequence neighborhood in the genome. However, because the number of different sequence neighborhoods goes as 2^*N*^, even for modest *N* there is not enough data to make accurate estimates.

To overcome this we utilize the principle of maximum entropy to calculate probability distributions, $P(\vec{\sigma }|c,d)$ and $P(\vec{\sigma }|d)$, that reproduce a few reliably estimated statistics of $\vec{\sigma }$ extracted from experimental Hi-C data [37, 38]. The statistics we use to constrain both of the above distributions are the average spin 〈*σ*_*k*_〉 at a given position *k* in the neighborhood (which represents the average chromatin state at that neighbourhood postion) and the average spin-spin correlation 〈*σ*_*k*_*σ*_*l*_〉 between sites *k* and *l* in the neighborhood (which represents the average correlation between the chromatin states at the two neighbourhood positions). The averages denoted by 〈⋅〉 are calculated over their respective ensembles of sequence neighbourhoods ${\left\{\vec{\sigma }\right\}}_{d}$ that surround pairs of sites at a distance of contact *d* = |*j* − *i*|. The ensembles for both distributions can be extracted by scrolling along the sequence of binary states in the genome. When characterizing $P(\vec{\sigma }|c,d)$, each neighbourhood has a weight equal to the number of contacts *n*_*ij*_ observed between sites *i* and *j*; when characterizing $P(\vec{\sigma }|d)$, each neighbourhood has a unit weight for every time it appears in the genome (as shown in Fig 1 and Materials and methods). With the above statistics as constraints, the maximum-entropy distribution can be found using the method of Lagrange multipliers [37–39] and has the form of a Boltzman distribution for the Ising model at *k*_*B*_*T* = 1,

$$\begin{array}{c} P\left(\vec{\sigma }|\cdot \right)=\frac{e^{\sum_{k}h_{k}\sigma_{k}+{\sum \sum }_{l>k}J_{kl}\sigma_{l}\sigma_{k}}}{Z(\vec{\sigma }|\cdot )}, \end{array}$$ (2)

where *h*_*k*_ and *J*_*kl*_ are Lagrange multipliers that constitute the fitting parameters of the model. The partition function $Z(\vec{\sigma }|\cdot )$ is a normalization constant obtained by summing the numerator of Eq 2 over all possible $\vec{\sigma }$ neighborhoods, $Z\left(\vec{\sigma }|\cdot \right)=\sum_{\vec{\sigma }}\text{exp}(\sum_{k}h_{k}\sigma_{k}+{\sum \sum }_{l>k}J_{kl}\sigma_{l}\sigma_{k})$. In the above, we use “|⋅” to summarize in a single notation two different conditions at each distance of contact, namely $P(\vec{\sigma }|c,d)$ and $P(\vec{\sigma }|d)$ (“⋅” substitutes “*c*, *d*” or “*d*”). For neighborhoods of size up to *N* ∼ 23, exact enumeration can be used to evaluate Eq 2 in minimal time. Beyond that, one would need to estimate this distribution by Monte Carlo sampling [40]. We have thus restricted our neighbourhoods to a maximum size *N* = 20 so that we can conveniently calculate Eq 2 by enumerating the 2^*N*^ possible neighbourhoods at a given *d* (see Materials and methods).

At a given genomic distance *d* the distribution in Eq 2 can be fit to reproduce the experimental statistics 〈*σ*_*k*_〉 and 〈*σ*_*k*_*σ*_*l*_〉 calculated from the sequence ensemble at that distance (see Materials and methods). We now detail how we applied this method to estimate these distributions and ultimately the contact probability, Eq 1, from real experimental sequence and structural data.

### Model parameters

As a test of the method, we used the measured structure and sequence data for *Drosophila Melanogaster*. For structure, we used a Hi-C contact map from *Drosophila* embryos, generated using sites that were 10 kilobasepair (kbp) in size [41]. For sequence, we used our own binary classification (spin-up or spin-down) of the measured *Drosophila* chromatin-associated factors at each 10 kbp site, which was based on the “chromatin colors” classification [11] (see Materials and methods). To avoid overfitting the model, we divided the data from *Drosophila* chromosomes 2 and 3 into a training set used for fitting and a test set for prediction (see Materials and methods).

**Fig 2:**
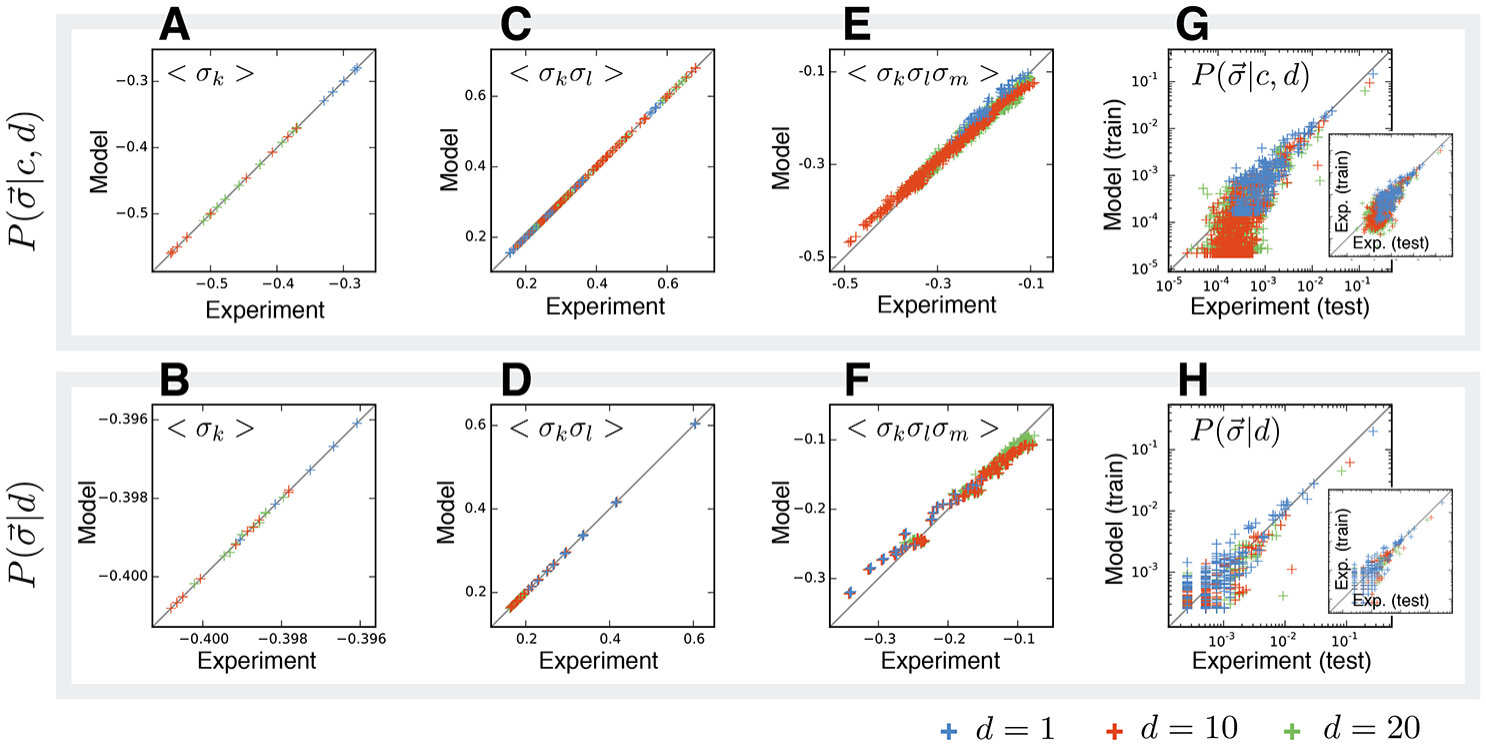
Spin statistics of model versus experiment. Spin statistics at three different contact distances, *d*. Top row is for models conditioned on contact, $P(\vec{\sigma }|c,d)$, whereas the bottom row is for models that are regardless of contact, $P(\vec{\sigma }|d)$. (A, B) Site average statistics, 〈*σ*_*k*_〉. (C, D) Pairwise correlation statistics, 〈*σ*_*l*_*σ*_*k*_〉. (E, F) Three body correlation statistics, 〈*σ*_*k*_*σ*_*l*_*σ*_*m*_〉. (G, H) The probability of $\vec{\sigma }$ from the model versus the observed frequency from contact maps (G) and from the genome (H). For reference the insert compares experimentally measured spin-vector probabilities from training versus testing data.

For genomic distances ranging from *d* = 10 kbp to 800 kbp in 10 kbp steps, we used the above maximum-entropy method to estimate the two conditional probability distributions, $P(\vec{\sigma }|c,d)$ and $P(\vec{\sigma }|d)$. Fig 2(A)–2(D) shows that the fitted distributions successfully predicted the experimental statistics 〈*σ*_*k*_〉 and 〈*σ*_*k*_*σ*_*l*_〉 on the test data. They also captured three-point correlations 〈*σ*_*k*_*σ*_*l*_*σ*_*m*_〉 (see Fig 2(E) and 2(F)) despite not having incorporated them into the fit. In Fig 2(G) and 2(H) we also see that the predicted sequence neighborhood probabilities $P(\vec{\sigma }|\cdot )$ from the model agreed with their frequencies as seen in the data. Thus these second-order maximum-entropy distributions seemed to be adequate approximations to the true distributions (first-order maximum-entropy distributions were also tested and failed to reproduce experimental statistics, see S3 Fig and S1 Text).

Inspection of the associated fit parameters *h*_*k*_ and *J*_*lk*_ as a function of genomic distance, *d*, showed that they naturally clustered into two groups (through both K-means and Principal Component Analysis), with a transition from one to the other occurring at *d* ≈ 390 kbp (see S4 Fig and S1 Text). Averaging the parameters within each group together we found that 〈*h*_*k*_〉 of the two sites in contact were positive (especially for the group above transition). Positive values for *h*_*k*_ favour the spin-up active/euchromatic state and thus, the above finding shows that contacting sites had a tendency to be in the such a state. Interestingly, the sites in between the contacting sites had an inactive/heterochromatic preference (i.e. negative values for 〈*h*_*k*_〉) in the group below the transition, and no chromatin preference in the group above. Therefore at short genomic separations, a heterochromatic region between contacting sites favored contact. We also found that the interaction terms 〈*J*_*lk*_〉 for the neighbors between contacting sites were ferromagnetic for the group below the transition (i.e. 〈*J*_*lk*_〉 > 0 which favors sites *l* and *k* to be in the same state) and were antiferromagnetic for the group above (i.e *J*_*k*_*l* < 0 which favours *l* and *k* to be in opposite states). Thus at short genomic distances, the sequence between the contacting sites was favored to be homogeneous, whereas at longer distances heterogeneity or a more random sequence seemed to be the case (see S4 Fig and S1 Text).

### Contact map prediction

**Fig 3:**
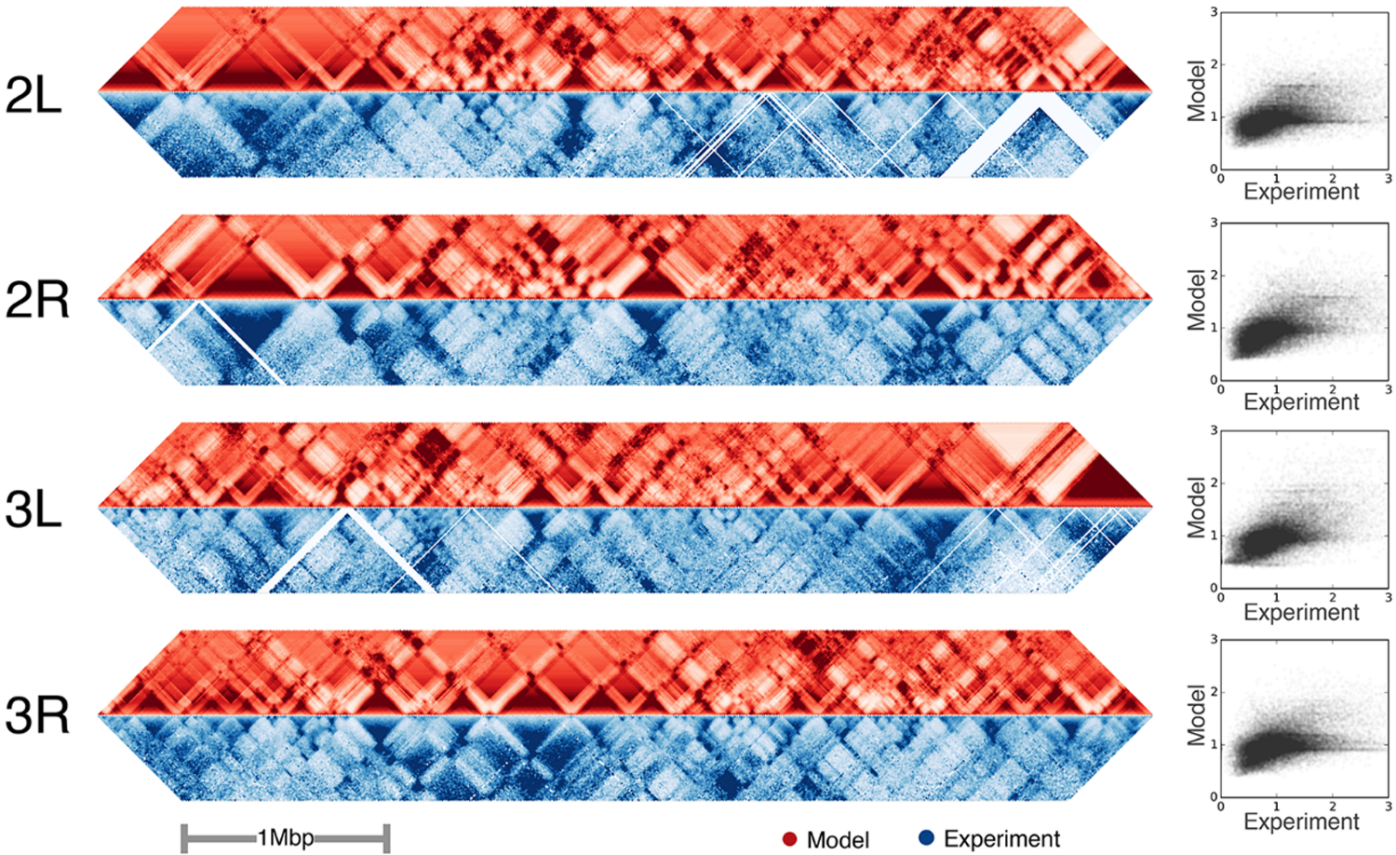
Contact prediction in test set. Diagonal elements of contact maps (shown as heat maps) from the 5 Mbp test regions situated at the ends of chromosomes 2 and 3 of *Drosophila melanogaster*. The maps span genomic distances from 10 kbp to 800 kpb for sites that are 10 kbp in size. (The midline of each contact map corresponds to *i* = *j*). Predicted contact maps (red heat maps) and the experimentally measured maps (blue heat maps) from Schuettengruber et al [41] Hi-C data. Both the experimental and predicted maps were distance normalized as described in the main text. The column on the right shows the predicted versus experimental contact likelihood for each pair of sites in the test data as a scatter plot.

We used the fitted maximum entropy distributions, $P(\vec{\sigma }|c,d)$ and $P(\vec{\sigma }|d)$ at each distance, *d*, along with Eq (1) to make predictions for distance-normalized contact maps $P\left(c|\vec{\sigma },d\right)/P\left(c|d\right)$ on the test data from *Drosophila* chromosomes 2 and 3 (red heat maps in Fig 3). Each (*i*, *j*) pair had an associated $\vec{\sigma }$ from which we could then evaluate Eq 1 using the fitted distributions. (We normalized by *P*(*c*|*d*) so as to remove the strong decay with distance of the probability of making a contact). These were then correlated to the normalized experimental Hi-C contact maps *n*_*ij*_/〈*n*(*d*)〉 of the test data (blue heat maps in Fig 3), where 〈*n*(*d*)〉 is the average number of Hi-C contact counts at a given distance *d*, that also decays strongly with distance. The correlations with the *Drosophila* chromosomes were 0.39 (chromosome 2L), 0.53 (chromosome 2R), 0.53 (chromosome 3L), and 0.40 (chromosome 3R). These correlations are remarkable given that only a binary model for the sequence was used. The areas of discrepancy in Fig 3 may be due to the fact that the binding factors were measured from different cell lines than the Hi-C ones, so it is possible that in those regions the actual underlying sequence that generated the observed contact counts may be different than what was used in making the prediction.

### Structural changes due to sequence mutation

The fitted maximum-entropy model that can predict contact maps from chromatin sequence provides an opportunity to also predict structural changes that might arise due to mutations in the underlying sequence of bound factors. We thus set to identify which genomic locations are expected to disrupt the local structure the most in the event that their chromatin state is flipped. This analysis was performed in genomic regions where the locally-predicted contact probabilities agreed the most with experimental measurements (correlation between predicted and experimental distance-normalized probabilities of contact *c* > 0.6). The chromatin state, *σ*, of each of these well-predicted locations was individually inverted and the correlation, *c*′, between the newly predicted probabilities and the experimental ones was calculated (see Materials and methods for details). We interpret the change in correlation Δ*c* = *c*′ − *c* to be a measure of structural-sensitivity to chromatin sequence mutations for that position.

**Fig 4:**
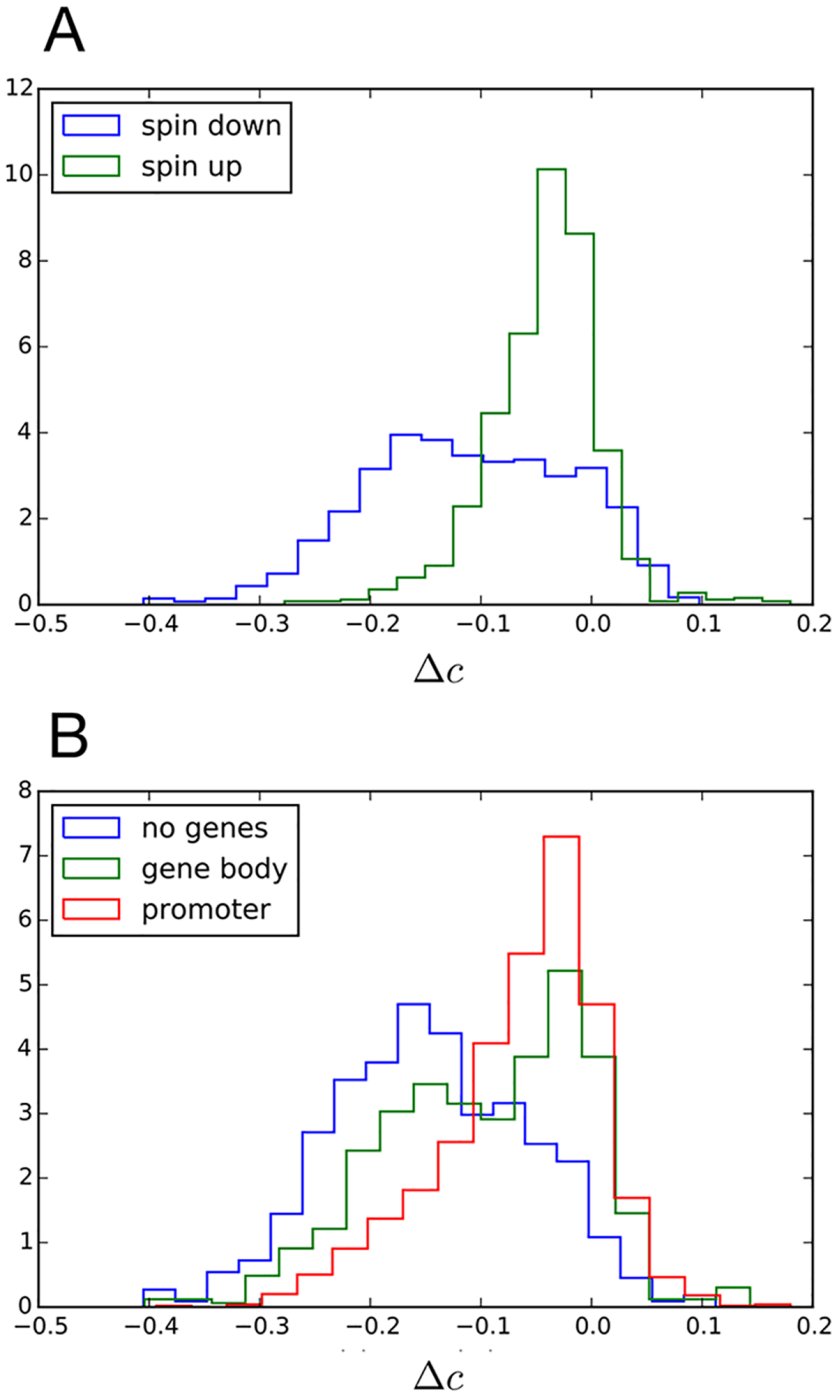
Structural changes by chromatin mutation. Single site chromatin mutations were performed by inverting the chromatin state of genomic locations where the correlation between the experimental and model-predicted probability of contact was initially high (correlation *c* > 0.6). The change in correlation with experimental probabilities after mutation Δ*c* was measured for different non-overlapping genomic classifications. (A) Spin-up and spin-down sites. (B) Sites that did not contain genes, sites that had promoter regions and “gene body” sites that contained genes but no promoters.

In Fig 4 we show histograms of Δ*c* for genomic locations categorized by genomic feature. Regardless of genomic feature, the vast majority of sites had a negative Δ*c* after their chromatin state was inverted, indicating that the local predicted structure tended to depart from the experimental structure when the sequence state was mutated. In Fig 4A we found that the inactive/heterochromatic (spin down) sites were significantly more structurally sensitive than the active/euchromatin (spin up) sites (average Δ*c* was -0.11 for spin-down whereas -0.04 for spin-up sites). In other words, a change from inactive to active DNA tended to produce a greater structural change than the opposite. In Fig 4B we classified genomic sites into three non-overlapping categories: sites where no genes were present, sites that contained gene promoters, and “gene body” sites corresponding to locations occupied by genes but with no promoters. We found that sites with no genes were the most structurally sensitive, followed by “gene body” sites and lastly, the least structurally sensitive were sites containing promoters (average Δ*c* was -0.15 for no genes, -0.10 for “gene body” and -0.06 for promoter sites). These findings highlight that non-coding regions of the genome have the greatest capacity to alter DNA structure, whereas mutations in gene rich regions are less likely to cause significant alterations to structure.

### Predicting sequence from structure

Here we show that it is possible to solve the inverse problem, namely, given a Hi-C map of contact counts, {*n*_*ij*_}, and a model for the probability of a sequence neighborhood $\vec{\sigma }$ to be in contact, determine the probability of each site *k* to be in a particular sequence state (*σ*_*k*_ = 1 or *σ*_*k*_ = −1). We denote this probability as *P*(*σ*_*k*_|{*n*_*ij*_}) where {*i*, *j*} is the set of all contacting pairs that contain genomic site *k* in their sequence neighborhood. By applying Bayes’ rule we can write it as

$$\begin{array}{c} P\left(\sigma_{k}|\left\{n_{ij}\right\}\right)=\frac{P\left(\left\{n_{ij}\right\}|\sigma_{k}\right)P\left(\sigma_{k}\right)}{P(\left\{n_{ij}\right\})}, \end{array}$$ (3)

where *P*(*σ*_*k*_) is a prior on the sequence state at site *k*, and *P*({*n*_*ij*_}) the probability of the data that simply acts as a normalization constant. As we show in S1 Text, Eq 3 can be rewritten as

$$\begin{array}{c} \begin{array}{c} P\left(\sigma_{k}|\left\{n_{ij}\right\}\right)=\frac{P{\left(\sigma_{k}\right)}^{1-M}}{P(\left\{n_{ij}\right\})}\prod_{i,j}\sum_{\vec{\sigma }}P\left(n_{ij}|\vec{\sigma },d\right)P\left(\vec{\sigma }|d\right)\delta_{\sigma_{k^{\prime }},\sigma_{k}}, \end{array} \end{array}$$ (4)

where $P(n_{ij}|\vec{\sigma },d)$ is the probability of observing a particular number of counts between sites *i* and *j* given their sequence neighborhood, $\vec{\sigma }$, *k*′ is the position of genomic site *k* in that neighborhood, and *M* is the the number of {*i*, *j*} pairs. We take $P(n_{ij}|\vec{\sigma },d)$ to be a Gaussian distribution $N(\lambda_{\vec{\sigma },d},\zeta_{\vec{\sigma },d}^{2})$ with mean equal to the average number of counts for a given sequence $\vec{\sigma }$, $\lambda_{\vec{\sigma },d}=\lambda \left(c|\vec{\sigma },d\right)$, which is proportional to the probability of contact $P(c|\vec{\sigma },d)$ that can be evaluated from Eq 1. The variance, $\zeta_{\vec{\sigma },d}^{2}$ can be estimated from the Hi-C data (see S1 Text for more details).

**Fig 5:**
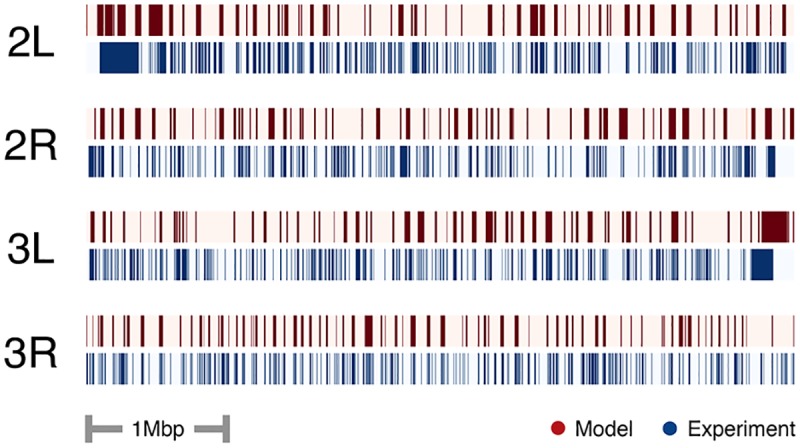
Prediction of bound factors from contact map. Sequence of bound factors as a function of site along the four 5 Mbp test regions at the end of chromosomes 2 and 3 in *Drosophila*. In blue are shown the locations of spin-up sequence sites, *σ*_*k*_ = 1 as classified from the experimental measurements of bound factors. The predicted probability of each site being spin-up, *σ*_*k*_ = 1 using the inverse method is shown in red.

Using just the observed Hi-C counts *n*_*ij*_ for the test data and our prior fitted maximum entropy models for $P(c|\vec{\sigma },d)$ and $P(\vec{\sigma }|d)$, we calculated the probability of each site in the test set being spin-up. Fig 5 shows this probability as a function of genomic location along the four test chromosome regions. Applying a threshold to these probabilities (*P*(*σ*_*k*_ = 1) = 0.5), we predicted a sequence with a percent agreement with the original test sequence of 78% (chromosome 2L), 72% (chromosome 2R), 77% (chromosome 3L) and 77% (chromosome 3R). Thus structure alone can yield an important amount of information about the underlying sequence of bound chromatin factors. We feel that this could have significant impact in determining important regions for regulating structure. In particular, if the maximum entropy model is reliably capturing the essential aspects that connect sequence to structure and if one believes that these aspects are conserved across different cell types, one could use a fitted model from one cell type to predict sequence in another for which only structural information is known.

## Discussion

Our results demonstrate that a model for the probability of contact between two genomic sites given just their neighborhood of bound factors can be estimated using a maximum-entropy method. The resulting model did well at predicting the contact map of a test set using only sequence information.

This model connects sequence to structure and thus offers the capability of testing the structural effect of mutating the chromatin sequence. Our analysis highlights that the alteration of DNA conformation by mutating chromatin sequence is particularly strong in sites containing no genes, while only minimal alterations to structure tend to occur when mutating active gene-rich regions.

Although we applied our fit model to data from *Drosophila* embryos, we envision that it could be useful in predicting contact maps from sequence data taken from other cell types from the same species (e.g. different developmental times or from mutated cell lines), or potentially other nearby species where the contact model might expect to hold.

We also showed how a fitted model connecting sequence to structure could be used to solve the inverse problem, namely that a sequence of bound factors could be predicted using just measured counts from a Hi-C contact map and a model for the probability of contact given sequence. One could imagine that making such sequence predictions from Hi-C measurements on individual cell lines may be more efficient than carrying out the potentially tens to hundreds of experiments needed to map the locations of all the bound factors.

The classification of the genome into chromatin states used to fit our model was based on the occupation patterns of specific binding factors rather than structure. Therefore, we consider the possibility that other classifications may have a greater ability to predict structure from sequence than the one presented. Future work will consider other possible ways of defining sequence states from data for the purpose of predicting structure through this maximum entropy scheme, which may help to further elucidate the biological mechanisms behind chromatin folding.

Despite the great variety of DNA-binding chromatin factors identified so far this method is able to reproduce DNA contact maps from binary classifications that group all of the observed marks into only two classes. It is therefore tempting to speculate that at the > 10 Kbp scale, once DNA-bound factors are positioned on the genome, they tend to behave alike when it comes to generating DNA contacts. If correct, this would imply that the utility of such variety of chromatin marks is not entirely related to stabilizing complex DNA structures where a large number of different architectural elements is required. Instead, our findings favor the idea that the observed diversity of chromatin states is primarily related to alternative functions other than creating complex large-scale structures, such as the programming of chromatin states at different developmental times, or genetic regulation.

Overall our model has the potential for efficiently making predictions about chromatin structure depending on whether such structural or sequence data are available.

## Materials and methods

### DNA contact maps

DNA contact maps were obtained from the publicly available Hi-C experiments done by Schuettengruber et al [41] (GSE61471), performed on 3000-4000 *Drosophila melanogaster* embryos, 16-18 hours after egg laying. The contact map is an array whose elements are the number of times a particular pair of genomic sites were found to be in contact. The contact map we used defined sites to be of a fixed size (10 kilobasepair (kbp)) and were non-overlapping across the genome. The counts, *n*_*ij*_, at a particular pair of sites *i* and *j* in the contact map were determined by counting up all sequenced pairs from the Hi-C measurement that fell into those sites. We normalized the contact map using the ICE method [6] so that total number of counts along each row across the contact map was the same. This removed potential biases between sites due to the Hi-C protocol.

### Binary chromatin sequence classification

For every 10 kbp site, *k*, in the genome, we classified the genomic distribution of its DNA-bound factors into two possible chromatin sequence states corresponding to bound states that are associated with euchromatin (active DNA regions), *σ*_*k*_ = 1, and heterochromatin (inactive DNA regions), *σ*_*k*_ = −1. In order to make these sequence assignments, we used the “chromatin colors” classification of the *Drosophila melanogaster* genome by Filion et al [11] (GSE22069) which categorizes the distribution of bound factors into five different bound states (black, blue, yellow, green and red) based on the DamID binding profiles of 53 different chromatin marks in the embryonic *Drosophila melanogaster* cell line Kc167 (8-12 hours). We then grouped the five “chromatin colors” into just a spin-up and spin-down state based on the biological functions of their associated DNA-bound factors: spin-down = black and blue, corresponding to inactive/heterochromatin and spin-up = yellow, green and red that correspond active/euchromatin. The color data gives the coordinates of the regions of the various colours. In order to assign a particular sequence state to each of our 10 kbp sites, we took the dominant color group (either active, or inactive) to be the unique sequence state of that site.

### Training and test data sets

For the purpose of validating our model, we divided the *Drosophila melanogaster* autosomal chromosomes, 2 (comes as 2L and 2R) and 3 (comes as 3L and 3R) into a training set and test set. The training dataset consisted of genomic locations 2L: 1Mbp-17.02Mbp, 2R: 1Mbp-15.15Mbp, 3L: 1Mbp-18.55Mbp, 3R: 1Mbp-21.91Mbp. The testing dataset consisted of genomic locations 2L: 17.02Mbp-22.02Mbp, 2R: 15.15Mbp-20.15Mbp, 3L: 18.55Mbp-23.55Mbp, 3R: 21.91Mbp-26.91Mbp.

### Extracting sequence neighborhoods

We constructed sets of sequence neighborhoods ${\left\{\vec{\sigma }\right\}}_{d}$ for genomic distances between pairs *d* = |*j* − *i*| ranging from *d* = 1 to *d* = 80 in units of the site size, 10 kbp. For a pair of sites *i* and *j* in the genome separated by *d*, we took the particular sequence neighborhood, $\vec{\sigma }$, to be the union of sequence windows centered around each site. Specifically, we took 10 sites centered around site *i* and 10 sites centered around site *j* to create a neighborhood size of *N* = 20 (see S2 Fig) where the given sequence neighborhood consists of sites $\vec{\sigma }=\left(\sigma_{i-4},\ldots ,\sigma_{i},\ldots ,\sigma_{i+5},\sigma_{j-5},\ldots ,\sigma_{j},\ldots ,\sigma_{j+4}\right)$. For distances, *d* < 11, it was not possible to take sequence windows of size 10 sites centered on each site, so for these cases, we defined the neighborhoods as $\vec{\sigma }=\left(\sigma_{i-4},\ldots ,\sigma_{i},\ldots ,\sigma_{j},\ldots ,\sigma_{j+4}\right)$ which had a size *N* = *d* + 9 (S2 Fig). For a fixed genomic distance between sites *d*, we then scrolled through all pairs of sites, {*i*, *j*} in both the training and test regions of the genome extracting their corresponding neighborhoods $\vec{\sigma }$. Since the directionality (left-right) of the sequence neighborhood should not influence the contact probability between *i* and *j*, we also added the inverted sequence for each $\vec{\sigma }$ to the collection of neighborhoods. Each genomic distance, *d*, thus had its own unique ensemble of sequence neighborhoods spin-vectors for both the training and test data.

### Calculating sequence neighborhood statistics

The maximum entropy distributions, $P(\vec{\sigma }|c,d)$ and $P(\vec{\sigma }|d)$, were constrained to match several statistics of the extracted sequence neighborhood ensembles. The statistics used are 〈*σ*_*k*_〉 and 〈*σ*_*l*_*σ*_*k*_〉. For the distribution $P(\vec{\sigma }|c,d)$, these statistics were calculated as weighted averages of the extracted ensemble of sequence neighborhoods, where for each neighborhood we used as a weight the number of times, *n*_*ij*_, that its contacting sites *i* and *j* were observed to be in contact. For $P(\vec{\sigma }|d)$ the above statistics were calculated as weighted averages where the weight of each sequence in the ensemble was equal to the number of times it appeared in the genome.

### Fitting the maximum entropy distributions

The parameters *h*_*k*_ and *J*_*lk*_ in

$$\begin{array}{c} P\left(\vec{\sigma }|\cdot \right)=\frac{e^{\sum_{k}h_{k}\sigma_{k}+{\sum \sum }_{l>k}J_{kl}\sigma_{l}\sigma_{k}}}{Z(\vec{\sigma }|\cdot )} \end{array}$$ (5)

were fit to reproduce the experimental statistics 〈*σ*_*k*_〉_exp_ and 〈*σ*_*k*_*σ*_*l*_〉_exp_ by following the method described by Tkačik et al. [42] that we summarize next. The term “|⋅” simultaneously denotes the two conditions that we characterized at each distance of contact: The probability of observing $\vec{\sigma }$ given contact $P(\vec{\sigma }|c,d)$ and the probability of observing $\vec{\sigma }$ regardless of contact $P(\vec{\sigma }|d)$. We therefore fit a set of parameters {*h*_*k*_, *J*_*lk*_} for each of the two conditions denoted by |⋅ and for each distance of contact *d*.

Briefly, given one of the two conditions above, we used the corresponding collection of spin vectors and their associated weights (as described in the previous section) to calculate the experimental statistics 〈*σ*_*k*_〉_exp_ and 〈*σ*_*k*_*σ*_*l*_〉_exp_. Then we implemented the following iterative scheme:

1. Start with random guess for *h*_*k*_ and *J*_*lk*_
2. Use Eq 5 to calculate $P(\vec{\sigma }|\cdot )$ for every possible $\vec{\sigma }$ and obtain 〈*σ*_*k*_〉_model_ and 〈*σ*_*k*_*σ*_*l*_〉_model_.
3. Check for convergence, |〈*σ*_*k*_〉_model_ − 〈*σ*_*k*_〉_exp_| < *ϵ* and |〈*σ*_*k*_*σ*_*l*_〉_model_ − 〈*σ*_*k*_*σ*_*l*_〉_exp_| < *ϵ*, where *ϵ* is the error tolerance (we used *ϵ* = 0.0001). If it hasn’t converged yet, continue.
4. Update *h*_*k*_ as Δ*h*_*k*_ = *α*(〈*σ*_*k*_〉_exp_ − 〈*σ*_*k*_〉_model_) and *J*_*lk*_ as Δ*J*_*lk*_ = *α*(〈*σ*_*k*_*σ*_*l*_〉_exp_ − 〈*σ*_*k*_*σ*_*l*_〉_model_), where *α* is a small learning rate (we used *α* = 0.025). Go back to step 2.

This scheme is guaranteed to converge towards a unique solution regardless of the initial choice of *h*_*k*_ and *J*_*lk*_.

### Structural changes due to sequence mutation

First, we identified the sites *k* for which the local predicted contact map was in good agreement with the experimental contact map. The local contact map consists of only the subset of genomic pairs of sites {*i*, *j*} whose contact probability $P(c|\vec{\sigma },d)$ is influenced by the state of site *k* (ie. *k* is one of the spins in the neighborhood $\vec{\sigma }$ of *i* and *j*). At each genomic site, we calculated the correlation *c* between the local predicted distance-normalized contacts, $P\left(c|\vec{\sigma },d\right)/P\left(c|d\right)$, and the local experimental distance-normalized contacts counts, *n*_*ij*_/〈*n*(*d*)〉), and selected the best correlating locations of the genome (*c* > 0.6) for further analysis. Next, at each of these selected locations the chromatin state was flipped and the correlation *c*′ between predicted and experimental distance-normalized counts was measured. We then defined Δ*c* = *c*′ − *c* as a measurement of structural disruption due to sequence mutation at a given site.

## Supporting information

S1 Text Supplementary methods. Supplementary methods are provided detailing the fit of a first-order maximum-entropy model, the inspection of model parameters and the prediction of chromatin states from contact maps.
(PDF)

S1 Fig The effect of neighboring chromatin on DNA contacts. Top scheme illustrates how that the probability of contact between two sites *i* and *j* may be altered if *i* interacts favorably with another neighboring site *k*: Some of the time *i* and *k* will be in contact, therefore increasing the probability of contact between *i* and *j*. The scatter plot shows experimental evidence of this effect by looking at sites bound by the favorably-interacting DNA binding protein BEAF. In the plot, *i* and *k* are bound by BEAF, and *j* is not bound by BEAF except for when *j* = *k*. The y-axis represents the observed probability of *i* and *j* being in contact with respect to the background probability of contact of two sites at a distance |*j* − *i*| apart (irrespectively of whether BEAF is present). The x-axis represents the distance between *i* and *j* divided by the distance between *k* and *i*. The line plot is an analytic fit to the data using an ideal chain as a model for the polymer with an energy of interaction *E* between *i* and *k* as a fit parameter.
(TIF)

S2 FigSchematic of how the neighborhoods $\vec{\sigma }$ of the contacting sites (*i*, *j*) were built from the genomic distribution of chromatin states.First, the genome was binned into 10Kbp sites that could either be in a spin-up or spin-down state. (A,B) If *d* = |*j* − *i*| < 12, we defined the neighborhoods as $\vec{\sigma }=\left(i-4,\ldots ,i,\ldots ,j,\ldots ,j+4\right)$. (C) For *d* ≥ 12 we defined the neighborhoods as the union of the ten neighbors surrounding both *i* and *j* $\vec{\sigma }=\left(i-4,\ldots ,i,\ldots ,i+5,j-5,j,\ldots ,j+4\right)$ thus keeping them at a maximum size of *N* = 20.(TIF)

S3 FigSpin statistics of first-order maximum entropy model versus experiment.Spin statistics at three different contact distances, *d*. Top row is for models conditioned on contact, $P(\vec{\sigma }|c,d)$, whereas the bottom row is for models that are regardless of contact, $P(\vec{\sigma }|d)$. (A, B) Site average statistics, 〈*σ*_*k*_〉. (C, D) Pairwise correlation statistics, 〈*σ*_*l*_*σ*_*k*_〉. (E, F) Three body correlation statistics, 〈*σ*_*k*_*σ*_*l*_*σ*_*m*_〉. (G, H) The probability of $\vec{\sigma }$ from the model versus the observed frequency from contact maps (G) and from the genome (H). For reference the insert compares experimentally measured spin-vector probabilities from training versus testing data.(TIF)

S4 FigInspection of model parameters.(A) Average number of structures (2^*S*^) stored in the probability distributions of neighbors given contact between *i* and *j*, $P(\vec{\sigma }|c,d)$, and irrespectively of contact, $P(\vec{\sigma }|d)$, as a function of distance of contact *d* = |*j* − *i*|. (B) Kullback-Liebler divergence between probability distribution of neighbors given contact and irrespectively of contact as a function of distance. (C) Kullback-Liebler divergence between the contacting distributions at different distances of contact minus Kullback-Liebler divergence between the background distributions: $\Delta D[P\left(\vec{\sigma }|c,d_{1}\right)||P\left(\vec{\sigma }|c,d_{2}\right)]=D[P\left(\vec{\sigma }|c,d_{1}\right)||P\left(\vec{\sigma }|c,d_{2}\right)]-D[P\left(\vec{\sigma }|d_{1}\right)||P\left(\vec{\sigma }|d_{2}\right)]$. Blue indicates low values whereas red indicates high values. (D) A vector concatenating the difference between energetic coefficients for neighborhoods in contact and background, $\Delta h_{k}^{d}=h_{k}^{c,d}-h_{k}^{bg,d}$ and $\Delta J_{kl}^{d}=J_{kl}^{c,d}-J_{kl}^{bg,d}$, was built at every distance of contact. K-means clustering of the set of coefficient vectors naturally separated them into two clusters, one for *d* < 390 Kbp and another for *d* ≥ 390 Kbp. (E) Average of energetic coefficient vectors in K-means cluster 1. Blue indicates negative values whereas red indicates positive values. (F) Average of energetic coefficient vectors in K-means cluster 2. Blue indicates negative values whereas red indicates positive values. (G) Principal Component Analysis was applied to the set of energetic coefficient vectors. PC1 separates vectors into two two clusters, delimited at 390 Kbp, the same distance that separates K-means clusters. (H) Coefficient vectors projected into PC1 highlight the differences between the coefficients in the two clusters. Blue indicates negative values whereas red indicates positive values.(TIF)

## References

[1] Gurudatta B, Corces VG. Chromatin insulators: lessons from the fly. Briefings in functional genomics & proteomics. 2009;8(4):276–282. doi: 10.1093/bfgp/elp032 19752045 10.1093/bfgp/elp032 PMC2742804

[2] Phillips-Cremins JE, Corces VG. Chromatin insulators: linking genome organization to cellular function. Molecular Cell. 2013;50(4):461–474. 23706817 10.1016/j.molcel.2013.04.018 PMC3670141

[3] Cavalli G, Misteli T. Functional implications of genome topology. Nature structural & molecular biology. 2013;20(3):290–299. doi: 10.1038/nsmb.2474 10.1038/nsmb.2474 PMC6320674 23463314

[4] De Laat W, Duboule D. Topology of mammalian developmental enhancers and their regulatory landscapes. Nature. 2013;502(7472):499–506. doi: 10.1038/nature12753 24153303 10.1038/nature12753

[5] Lieberman-Aiden E, van Berkum NL, Williams L, Imakaev M, Ragoczy T, Telling A, et al Comprehensive mapping of long-range interactions reveals folding principles of the human genome. Science. 2009;326(5950):289–293. doi: 10.1126/science.1181369 19815776 10.1126/science.1181369 PMC2858594

[6] Imakaev M, Fudenberg G, McCord RP, Naumova N, Goloborodko A, Lajoie BR, et al Iterative correction of Hi-C data reveals hallmarks of chromosome organization. Nature methods. 2012;9(10):999–1003. doi: 10.1038/nmeth.2148 22941365 10.1038/nmeth.2148 PMC3816492

[7] Dixon JR, Selvaraj S, Yue F, Kim A, Li Y, Shen Y, et al Topological domains in mammalian genomes identified by analysis of chromatin interactions. Nature. 2012;485(7398):376–380. doi: 10.1038/nature11082 22495300 10.1038/nature11082 PMC3356448

[8] Roy S, Ernst J, Kharchenko PV, Kheradpour P, Negre N, Eaton ML, et al Identification of functional elements and regulatory circuits by Drosophila modENCODE. Science. 2010;330(6012):1787–1797. doi: 10.1126/science.1198374 21177974 10.1126/science.1198374 PMC3192495

[9] Thurman RE, Day N, Noble WS, Stamatoyannopoulos JA. Identification of higher-order functional domains in the human ENCODE regions. Genome research. 2007;17(6):917–927. doi: 10.1101/gr.6081407 17568007 10.1101/gr.6081407 PMC1891350

[10] Lian H, Thompson WA, Thurman R, Stamatoyannopoulos JA, Noble WS, Lawrence CE. Automated mapping of large-scale chromatin structure in ENCODE. Bioinformatics. 2008;24(17):1911–1916. doi: 10.1093/bioinformatics/btn335 18591192 10.1093/bioinformatics/btn335 PMC2519158

[11] Filion GJ, van Bemmel JG, Braunschweig U, Talhout W, Kind J, Ward LD, et al Systematic protein location mapping reveals five principal chromatin types in Drosophila cells. Cell. 2010;143(2):212–224. doi: 10.1016/j.cell.2010.09.009 20888037 10.1016/j.cell.2010.09.009 PMC3119929

[12] Ernst J, Kellis M. Discovery and characterization of chromatin states for systematic annotation of the human genome. Nature biotechnology. 2010;28(8):817–825. doi: 10.1038/nbt.1662 20657582 10.1038/nbt.1662 PMC2919626

[13] Kharchenko PV, Alekseyenko AA, Schwartz YB, Minoda A, Riddle NC, Ernst J, et al Comprehensive analysis of the chromatin landscape in Drosophila melanogaster. Nature. 2011;471(7339):480–485. doi: 10.1038/nature09725 21179089 10.1038/nature09725 PMC3109908

[14] Rosa A, Becker NB, Everaers R. Looping probabilities in model interphase chromosomes. Biophysical journal. 2010;98(11):2410–2419. doi: 10.1016/j.bpj.2010.01.054 20513384 10.1016/j.bpj.2010.01.054 PMC2877331

[15] Vettorel T, Grosberg AY, Kremer K. Statistics of polymer rings in the melt: a numerical simulation study. Physical biology. 2009;6(2):025013 doi: 10.1088/1478-3975/6/2/025013 19571364 10.1088/1478-3975/6/2/025013

[16] Halverson JD, Lee WB, Grest GS, Grosberg AY, Kremer K. Molecular dynamics simulation study of nonconcatenated ring polymers in a melt. I. Statics. The Journal of chemical physics. 2011;134(20):204904 doi: 10.1063/1.3587138 21639474 10.1063/1.3587137

[17] Mirny LA. The fractal globule as a model of chromatin architecture in the cell. Chromosome research. 2011;19(1):37–51. doi: 10.1007/s10577-010-9177-0 21274616 10.1007/s10577-010-9177-0 PMC3040307

[18] Wong H, Marie-Nelly H, Herbert S, Carrivain P, Blanc H, Koszul R, et al A predictive computational model of the dynamic 3D interphase yeast nucleus. Current biology. 2012;22(20):1881–1890. doi: 10.1016/j.cub.2012.07.069 22940469 10.1016/j.cub.2012.07.069

[19] Tjong H, Gong K, Chen L, Alber F. Physical tethering and volume exclusion determine higher-order genome organization in budding yeast. Genome research. 2012;22(7):1295–1305. doi: 10.1101/gr.129437.111 22619363 10.1101/gr.129437.111 PMC3396370

[20] Ganai N, Sengupta S, Menon GI. Chromosome positioning from activity-based segregation. Nucleic acids research. 2014; p. gkt1417. doi: 10.1093/nar/gkt1417 10.1093/nar/gkt1417 PMC3985638 24459132

[21] Bohn M, Heermann DW, van Driel R. Random loop model for long polymers. Physical Review E. 2007;76(5):051805 doi: 10.1103/PhysRevE.76.051805 10.1103/PhysRevE.76.051805 18233679

[22] Jost D, Carrivain P, Cavalli G, Vaillant C. Modeling epigenome folding: formation and dynamics of topologically associated chromatin domains. Nucleic acids research. 2014; p. gku698. doi: 10.1093/nar/gku698 10.1093/nar/gku698 PMC4150797 25092923

[23] Sanborn AL, Rao SS, Huang SC, Durand NC, Huntley MH, Jewett AI, et al Chromatin extrusion explains key features of loop and domain formation in wild-type and engineered genomes. Proceedings of the National Academy of Sciences. 2015;112(47):E6456–E6465. doi: 10.1073/pnas.1518552112 10.1073/pnas.1518552112 PMC4664323 26499245

[24] Di Pierro M, Zhang B, Aiden EL, Wolynes PG, Onuchic JN. Transferable model for chromosome architecture. Proceedings of the National Academy of Sciences. 2016; p. 201613607. doi: 10.1073/pnas.1613607113 10.1073/pnas.1613607113 PMC5087044 27688758

[25] Di Pierro M, Cheng RR, Aiden EL, Wolynes PG, Onuchic JN. De novo prediction of human chromosome structures: Epigenetic marking patterns encode genome architecture. Proceedings of the National Academy of Sciences. 2017; p. 201714980. doi: 10.1073/pnas.1714980114 10.1073/pnas.1714980114 PMC5699090 29087948

[26] Michieletto D, Orlandini E, Marenduzzo D. Polymer model with Epigenetic Recoloring Reveals a Pathway for the de novo Establishment and 3D Organization of Chromatin Domains. Physical Review X. 2016;6(4):041047 doi: 10.1103/PhysRevX.6.041047

[27] Mukhopadhyay S, Schedl P, Studitsky VM, Sengupta AM. Theoretical analysis of the role of chromatin interactions in long-range action of enhancers and insulators. Proceedings of the National Academy of Sciences of the United States of America. 2011;108(50):19919–19924. doi: 10.1073/pnas.1103845108 22123989 10.1073/pnas.1103845108 PMC3250180

[28] Michieletto D, Marenduzzo D, Wani AH. Chromosome-wide simulations uncover folding pathway and 3D organization of interphase chromosomes. arXiv preprint arXiv:160403041. 2016;.

[29] Barbieri M, Chotalia M, Fraser J, Lavitas LM, Dostie J, Pombo A, et al Complexity of chromatin folding is captured by the strings and binders switch model. Proceedings of the National Academy of Sciences. 2012;109(40):16173–16178. doi: 10.1073/pnas.1204799109 10.1073/pnas.1204799109 PMC3479593 22988072

[30] Brackley CA, Taylor S, Papantonis A, Cook PR, Marenduzzo D. Nonspecific bridging-induced attraction drives clustering of DNA-binding proteins and genome organization. Proceedings of the National Academy of Sciences. 2013;110(38):E3605–E3611. doi: 10.1073/pnas.1302950110 10.1073/pnas.1302950110 PMC3780866 24003126

[31] Benedetti F, Dorier J, Burnier Y, Stasiak A. Models that include supercoiling of topological domains reproduce several known features of interphase chromosomes. Nucleic acids research. 2013;42(5):2848–2855. doi: 10.1093/nar/gkt1353 24366878 10.1093/nar/gkt1353 PMC3950722

[32] Brackley C, Johnson J, Kelly S, Cook P, Marenduzzo D. Binding of bivalent transcription factors to active and inactive regions folds human chromosomes into loops, rosettes and domains. arXiv preprint arXiv:151101848. 2015;. 10.1093/nar/gkw135 PMC4856988 27060145

[33] Brackley CA, Babbs C, Marenduzzo D, Waithe D, Davies J, Brown JM, et al Predicting the three-dimensional folding of cis-regulatory regions in mammalian genomes using bioinformatic data and polymer models. Genome biology. 2016;17(1):59 doi: 10.1186/s13059-016-0909-0 27036497 10.1186/s13059-016-0909-0 PMC4815170

[34] Sexton T, Yaffe E, Kenigsberg E, Bantignies F, Leblanc B, Hoichman M, et al Three-dimensional folding and functional organization principles of the Drosophila genome. Cell. 2012;148(3):458–472. doi: 10.1016/j.cell.2012.01.010 22265598 10.1016/j.cell.2012.01.010

[35] Saeed S, Farré P, Olivier C, Eldon E. Probing long-range interactions by extracting free energies from genome-wide chromosome conformation capture data. BMC Bioinformatics. 2015;16:171 doi: 10.1186/s12859-015-0584-2 26001583 10.1186/s12859-015-0584-2 PMC4492175

[36] Vogelmann J, Valeri A, Guillou E, Cuvier O, Nollmann M. Roles of chromatin insulator proteins in higher-order chromatin organization and transcription regulation. Nucleus. 2011;2(5):358–369. doi: 10.4161/nucl.2.5.17860 21983085 10.4161/nucl.2.5.17860 PMC3796873

[37] Jaynes ET. Information theory and statistical mechanics. Physical review. 1957;106(4):620 doi: 10.1103/PhysRev.106.620

[38] Jaynes ET. Information theory and statistical mechanics. II. Physical review. 1957;108(2):171 doi: 10.1103/PhysRev.108.171

[39] Tkačik G, Marre O, Amodei D, Schneidman E, Bialek W, Berry MJ II. Searching for collective behavior in a large network of sensory neurons. PLoS Comput Biol. 2014;10(1):e1003408 doi: 10.1371/journal.pcbi.1003408 24391485 10.1371/journal.pcbi.1003408 PMC3879139

[40] Broderick T, Dudik M, Tkacik G, Schapire RE, Bialek W. Faster solutions of the inverse pairwise Ising problem. arXiv preprint arXiv:07122437. 2007;.

[41] Schuettengruber B, Elkayam NO, Sexton T, Entrevan M, Stern S, Thomas A, et al Cooperativity, specificity, and evolutionary stability of Polycomb targeting in Drosophila. Cell reports. 2014;9(1):219–233. doi: 10.1016/j.celrep.2014.08.072 25284790 10.1016/j.celrep.2014.08.072

[42] Tkacik G, Schneidman E, Berry I, Michael J, Bialek W. Ising models for networks of real neurons. arXiv preprint q-bio/0611072. 2006;.

