# Supplementary material for: A maximum-entropy model for predicting chromatin contacts
Source: PLoS Comput Biol. 2018 Feb 5;14(2):e1005956. doi: 10.1371/journal.pcbi.1005956 (PMC5814105; doi:10.1371/journal.pcbi.1005956)
Supplement: S1 Text — Supplementary methods are provided detailing the fit of a first-order maximum-entropy model, the inspection of model parameters and the prediction of chromatin states from contact maps. (PDF) [file pcbi.1005956.s001.pdf]

A maximum-entropy model for predicting chromatin contacts. Supplementary Material.

Pau Farré [1]◑, Eldon Emberly[1]◑,

1 Department of Physics, Simon Fraser University, Burnaby, BC V5A1S6, Canada

◑These authors contributed equally to this work.
¤Current Address: Department of Physics, Simon Fraser University, Burnaby, BC V5A1S6, Canada
* pfarrepe@sfu.ca

First-order maximum-entropy model

Here we present a first-order maximum-entropy model only constrained to reproduce one-spin statistics $\langle\sigma_k\rangle$ of the experimental distributions of neighborhoods $\vec{\sigma}$. Similarly to the second-order model presented in the main text, the first-order maximum-entropy distribution can be derived using the method of Lagrange multipliers [1–3] and has the following form:

$$P(\vec{\sigma}|\cdot) = \frac{e^{\sum_k h_k \sigma_k}}{Z(\vec{\sigma}|\cdot)}, \tag{1}$$

where h_k are Lagrange multipliers that constitute the fitting parameters of the model. The partition function $Z(\vec{\sigma}|\cdot)$ is obtained by summing the numerator over all possible $\vec{\sigma}$ neighborhoods. (In the above, we use "$|\cdot$" to summarize that we were fitting two different conditions, namely $P(\vec{\sigma}|c,d)$ and $P(\vec{\sigma}|d)$).

By noting that $Z(\vec{\sigma}|\cdot) = \prod_k e^{h_k} + e^{-h_k}$, we can rewrite Eq. 1 as the product of terms that only depend on k

$$P(\vec{\sigma}|\cdot) = \prod_k \frac{e^{h_k \sigma_k}}{e^{h_k} + e^{-h_k}}. \tag{2}$$

The terms in the product are normalized to unity and thus find that $P(\vec{\sigma}|\cdot)$ simply becomes the product of the independent probabilities for each σ_k in $\vec{\sigma}$,

$$P(\sigma_k|\cdot) = \frac{e^{h_k \sigma_k}}{e^{h_k} + e^{-h_k}}. \tag{3}$$

The value of h_k that matches the $\langle\sigma_k\rangle$ statistic can be found by solving

$$\langle\sigma_k\rangle = \sum_{\sigma_k} \sigma_k P(\sigma_k|\cdot), \tag{4}$$

which gives

$$h_k = \frac{1}{2}\log\frac{1+\langle\sigma_k\rangle}{1-\langle\sigma_k\rangle}. \tag{5}$$

S4 Fig.(A,B) shows that the distributions calculated from Eq. 3 successfully predicted the experimental statistics $\langle \sigma_k \rangle$ on the test data. However, this first-order maximum entropy distributions failed to capture both the second- (S4 Fig.(C,D)) and third- (S4 Fig.(E,F)) order statistics that were not incorporated into the fit. In S4 Fig.(G and H) we also see that the predicted sequence neighborhood probabilities $P(\vec{\sigma}|\cdot)$ from the model did not agree with their frequencies as seen in the data.

We thus conclude that the first-order maximum-entropy distribution in Eq. 3 is not a good-enough description of the experimental distributions of $\vec{\sigma}$ and further-order moment distributions need to be considered.

Inspection of model parameters

For each distance of contact $d = |j - i|$ we obtained two sets of parameters for $P(\vec{\sigma}|\cdot)$, one describing the probability of a given neighborhood $\vec{\sigma}$ given contact between i and j, $P(\vec{\sigma}|c,d) = f(h_k^{c,d}, J_{kl}^{c,d})$, and another set describing the probability of a given neighborhood regardless of contact (which we term here as *background*), $P(\vec{\sigma}|d) = f(h_k^{bg,d}, J_{kl}^{bg,d})$.

S5 Fig.A shows that the Shannon entropy [4],

$$S[P(\vec{\sigma}|\cdot)] = -\sum_{\vec{\sigma}} P(\vec{\sigma}|\cdot)\log_2 P(\vec{\sigma}|\cdot), \tag{6}$$

of the distribution sequences regardless of contact was greater than the entropy of the distribution of contacts at short distances ($d < 330$ Kbp) and the opposite happened for longer distances. In particular, the plot displays the average number of neighborhood configurations encoded in the probability distributions, 2^S.

S5 Fig.B displays the Kullback-Leibler (K-L) divergence between neighborhoods given contacts and background neighborhoods at each distance d,

$$D[P(\vec{\sigma}|c,d)||P(\vec{\sigma}|d)] = \sum_{\vec{\sigma}} P(\vec{\sigma}|c,d)\log_2 \frac{P(\vec{\sigma}|c,d)}{P(\vec{\sigma}|d)}, \tag{7}$$

that can be interpreted as a distance between the two probability neighborhood distributions, or the information gain when including information about contacts into the background distribution of neighbors, therefore going from $P(\vec{\sigma}|d)$ to $P(\vec{\sigma}|c,d)$. This quantity diminished with distance and saturated at its lowest value at a distance of ~ 300 Kbp.

We then explored the similarity between the distributions of contacting neighborhoods at different distances. Specifically, we subtracted the K-L divergence of the background from the K-L divergence of the contacting distributions (correcting for background sequence effects),

$$\begin{aligned} \Delta D[P(\vec{\sigma}|c,d_1)||P(\vec{\sigma}|c,d_2)] = \\ D[P(\vec{\sigma}|c,d_1)||P(\vec{\sigma}|c,d_2)] \\ -D[P(\vec{\sigma}|d_1)||P(\vec{\sigma}|d_2)]. \end{aligned} \tag{8}$$

S5 Fig.C we observe two regimes of similarity between the distributions at different distances, one for $d_1, d_2 < 390$ Kbp and another for $d_1, d_2 \geq 390$, where $\Delta D[P(\vec{\sigma}|c,d_1)||P(\vec{\sigma}|c,d_2)]$ takes the lowest values (blue denotes low ΔD whereas red corresponds to high ΔD).

We further compared models at different distances by analyzing the similarity of the energetic coefficients of enrichment defined as $\Delta h_k^d = h_k^{c,d} - h_k^{bg,d}$ and

$\Delta J_{kl}^d = J_{kl}^{c,d} - J_{kl}^{bg,d}$ since these were the parameters involved in the distance-normalized contacts as it can be derived from Eqs. 1 and 2 in the main text:

$$\begin{aligned}\frac{P(c|\vec{\sigma},d)}{P(c|d)} &= \frac{P(\vec{\sigma}|c,d)}{P(\vec{\sigma}|d)} \\ &= \frac{Z^{bg,d}}{Z^{c,d}} e^{-\sum_k \Delta h_k^d \sigma_k - \sum \sum_{l>k} \Delta J_{kl}^d \sigma_l \sigma_k}\end{aligned} \tag{9}$$

For every distance of contact, the parameters Δh_k^d and ΔJ_{kl}^d were concatenated into a vector, and the set of vectors corresponding to all distances was then clustered into two groups with K-means [5]. The coefficient vectors naturally separated at the distance of contact of 390 Kbp (S5 Fig.D). In S5 Fig.E and S5 Fig.F we show the average energetic coefficients of enrichment of the two K-means clusters, which differ both in Δh_k and ΔJ_{kl}.

In addition, we applied Principal Component Analysis (PCA) [6, 7] to the same set of coefficient vectors as above, and the first principal component (PC1) clearly separated the same clusters previously found by K-means delimited at a distance of contact of 390 Kbp (S5 Fig.G). PC1 shows positive scores for the shorter distances of contact ($d <$ 390Kbp) and negative scores for the longer distances of contact ($d \geq 390$ Kbp). Therefore, projecting the vectors of coefficients onto PC1, we found how the short-distance cluster differs from the long-distance cluster (S5 Fig.H), which corresponded to an increase of ferromagnetic interactions between the sites situated inside the loop.

Predicting sequence given structure

Given fitted maximum entropy distributions, $P(\vec{\sigma}|c,d)$ and $P(\vec{\sigma}|d)$, over a range of genomic distances d, we now work out how to solve the inverse problem, namely finding the probability of a genomic site k being in a particular binary state σ_k, given only structural data from a set of Hi-C counts $\{n_{ij}\}$. We denote this probability by $P(\sigma_k|\{n_{ij}\})$, where $\{n_{ij}\}$ is the set of counts between all (i,j) pairs of sites considered to be neighbors of k in our model.

As in the main text, Bayes' theorem gives

$$P(\sigma_k|\{n_{ij}\}) = \frac{P(\{n_{ij}\}|\sigma_k)P(\sigma_k)}{P(\{n_{ij}\})}, \tag{10}$$

where $P(\{n_{ij}\}|\sigma_k) = \prod_{ij} P(n_{ij}|\sigma_k,d)$. $P(n_{ij}|\sigma_k,d)$ is the probability of observing exactly n_{ij} contact counts between a pair of sites a distance $d = |j-i|$ apart given that the genomic site k in their sequence neighborhood is in a particular state, σ_k. (Note that d is a redundant variable whenever i and j are specified, ie. $P(n_{ij}|\sigma_k) = P(n_{ij}|\sigma_k,d)$. We nevertheless introduce it here for consistency with the rest of our notation). $P(\sigma_k)$ is the prior on σ_k and is the probability for site k to be in one of the two states (here we take it to be a constant over the genome, with the same value as measured in the training set $P(\sigma_k = 1) = 0.31$). $P(\{n_{ij}\})$ is simply a normalization constant and is found by summing the numerator over σ_k. Rewriting Eq. (10), we have,

$$P(\sigma_k|\{n_{ij}\}) = \frac{P(\sigma_k)}{P(\{n_{ij}\})} \prod_{ij} P(n_{ij}|\sigma_k,d). \tag{11}$$

Using k' to label the position that the genomic site k takes in the particular neighborhood of (i,j), $\vec{\sigma} = \{\sigma_1, \cdots, \sigma_{k'}, \cdots, \sigma_N\}$, and considering $P(\sigma_k|d) = P(\sigma_k)$ we

then rewrite $P(n_{ij}|\sigma_k, d)$ as

$$\begin{aligned} P(n_{ij}|\sigma_k, d) &= \frac{P(n_{ij}, \sigma_k|d)}{P(\sigma_k|d)} \\ &= \frac{P(n_{ij}, \sigma_k|d)}{P(\sigma_k)} \\ &= \sum_{\vec{\sigma}} \frac{P(n_{ij}, \vec{\sigma}, \sigma_k|d)}{P(\sigma_k)} \\ &= \sum_{\vec{\sigma}} \frac{P(n_{ij}, \vec{\sigma}|d)}{P(\sigma_k)} \delta_{\sigma_k, \sigma_{k'}} \\ &= \sum_{\vec{\sigma}} \frac{P(n_{ij}|\vec{\sigma}, d) P(\vec{\sigma}|d)}{P(\sigma_k)} \delta_{\sigma_k, \sigma_{k'}} \end{aligned} \tag{12}$$

where the Kronecker delta $\delta_{\sigma_k, \sigma_{k'}}$ ensures that we sum all possible sequences of the neighborhood $\vec{\sigma}$ that have genomic site k held fixed in a particular state σ_k.

Next, by combining Eqs. 11 and 12, we obtain

$$P(\sigma_k|\{n_{ij}\}) = \frac{P(\sigma_k)^{1-M}}{P(\{n_{ij}\})} \prod_{ij} \sum_{\vec{\sigma}} P(n_{ij}|\vec{\sigma}, d) P(\vec{\sigma}|d) \delta_{\sigma_{k'}, \sigma_k}, \tag{13}$$

where M is the number of (i, j) pairs that have sequence neighborhoods that contain genomic site k. The distribution, $P(\vec{\sigma}|d)$ is taken to be the fitted maximum entropy distribution at a distance d. We assume that the probability of observing n_{ij} Hi-C counts given a sequence state $\vec{\sigma}$, $P(n_{ij}|\vec{\sigma}, d)$, is as a Gaussian distribution $\mathcal{N}(\lambda_{\vec{\sigma},d}, \zeta^2_{\vec{\sigma},d})$ with a mean number of counts, $\lambda_{\vec{\sigma},d}$, proportional to the fitted probability of contact for the given sequence neighborhood $\vec{\sigma}$,

$$\lambda_{\vec{\sigma},d} = \lambda(c|\vec{\sigma}, d) = KP(c|\vec{\sigma}, d) = \frac{P(\vec{\sigma}|c, d)\lambda(c|d)}{P(\vec{\sigma}|d)}, \tag{14}$$

where K is a constant that depends on experimental details such as the number of cells used and the efficiency of contact detection, and $\lambda(c|d) = KP(c|d) = \langle n(d) \rangle$ is the experimental average of Hi-C counts at a distance d. With this and the two fitted maximum entropy distributions, we can calculate the mean number of counts for a given sequence neighborhood from Eq. 14. The variance $\zeta^2_{\vec{\sigma},d}$ is sampled from the train set as a function of λ. Specifically, we calculated the rates λ_{ij} associated to all Hi-C counts n_{ij} from the train set. Then, for various values of λ we collected the Hi-C counts n_{ij} that our model had assigned a rate λ_{ij} between $0.9 \times \lambda$ and $1.1 \times \lambda$. Lastly, we calculated the variance of the rate-associated counts $\zeta^2(\lambda)$ and fitted a polynomial curve to it (we found $\zeta^2 \approx \lambda^2$).

Everything in Eq. 13 is now determined and so the probability of a particular sequence state (either $\sigma_k = 1$ or $\sigma_k = -1$) at every site k can be calculated if given a Hi-C contact map, $\{n_{ij}\}$.

References

1. Jaynes ET. Information theory and statistical mechanics. Physical review. 1957;106(4):620.
2. Jaynes ET. Information theory and statistical mechanics. II. Physical review. 1957;108(2):171.

3. Tkačik G, Marre O, Amodei D, Schneidman E, Bialek W, Berry II MJ. Searching for collective behavior in a large network of sensory neurons. PLoS Comput Biol. 2014;10(1):e1003408.

4. Shannon CE. A mathematical theory of communication. ACM SIGMOBILE Mobile Computing and Communications Review. 2001;5(1):3–55.

5. Steinhaus H. Sur la division des corp materiels en parties. Bull Acad Polon Sci. 1956;1(804):801.

6. Pearson K. LIII. On lines and planes of closest fit to systems of points in space. The London, Edinburgh, and Dublin Philosophical Magazine and Journal of Science. 1901;2(11):559–572.

7. Hotelling H. Analysis of a complex of statistical variables into principal components. Journal of educational psychology. 1933;24(6):417.
