# Supplementary figures and images for: A maximum-entropy model for predicting chromatin contacts
Source: PLoS Comput Biol. 2018 Feb 5;14(2):e1005956. doi: 10.1371/journal.pcbi.1005956 (PMC5814105; doi:10.1371/journal.pcbi.1005956)

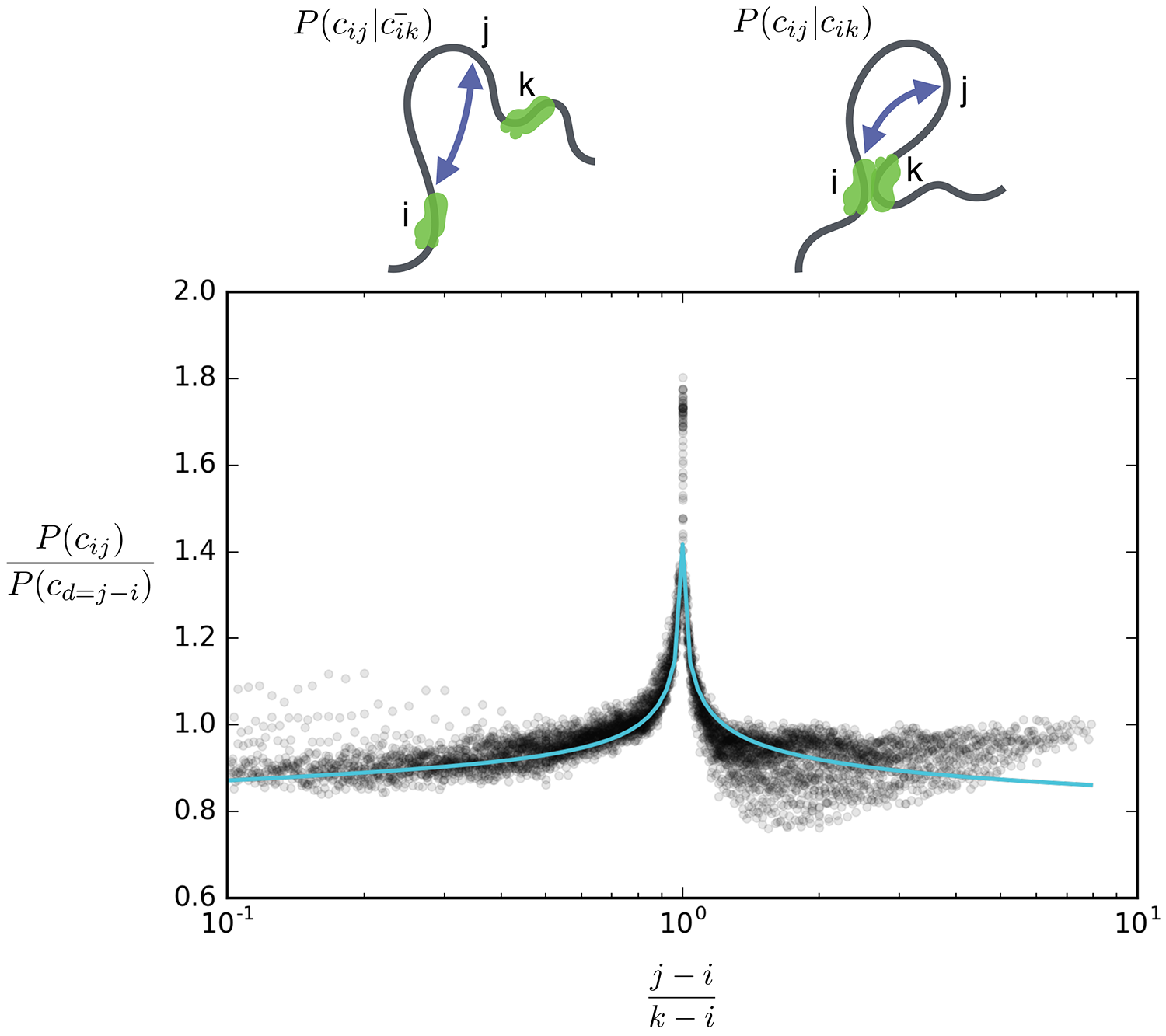

Supplement: S1 Fig — Top scheme illustrates how that the probability of contact between two sites i and j may be altered if i interacts favorably with another neighboring site k: Some of the time i and k will be in contact, therefore increasing the probability of contact between i and j. The scatter plot shows experimental evidence of this effect by looking at sites bound by the favorably-interacting DNA binding protein BEAF. In the plot, i and k are bound by BEAF, and j is not bound by BEAF except for when j = k. The y-axis represents the observed probability of i and j being in contact with respect to the background probability of contact of two sites at a distance |j − i| apart (irrespectively of whether BEAF is present). The x-axis represents the distance between i and j divided by the distance between k and i. The line plot is an analytic fit to the data using an ideal chain as a model for the polymer with an energy of interaction E between i and k as a fit parameter. (TIF) [file pcbi.1005956.s002.tif]

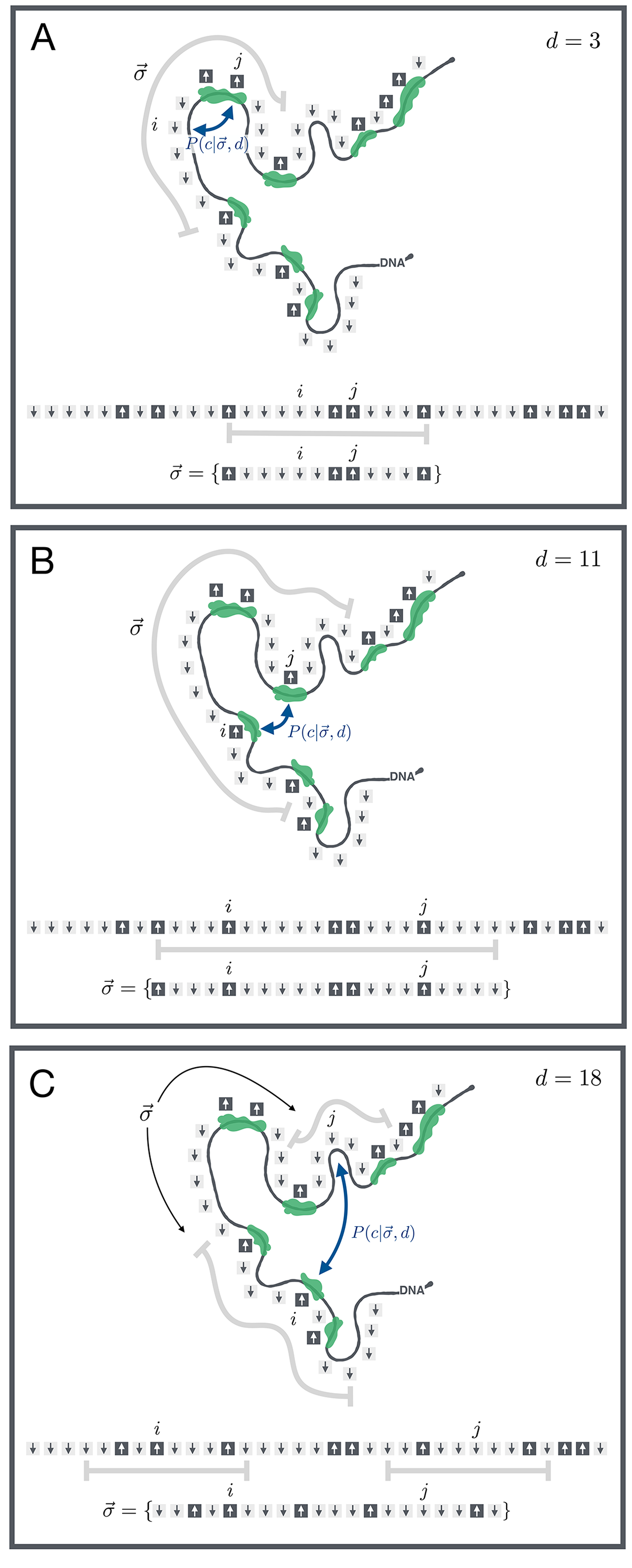

Supplement: S2 Fig — First, the genome was binned into 10Kbp sites that could either be in a spin-up or spin-down state. (A,B) If d = |j − i| < 12, we defined the neighborhoods as σ→=(i-4,…,i,…,j,…,j+4). (C) For d ≥ 12 we defined the neighborhoods as the union of the ten neighbors surrounding both i and j σ→=(i-4,…,i,…,i+5,j-5,j,…,j+4) thus keeping them at a maximum size of N = 20. (TIF) [file pcbi.1005956.s003.tif]

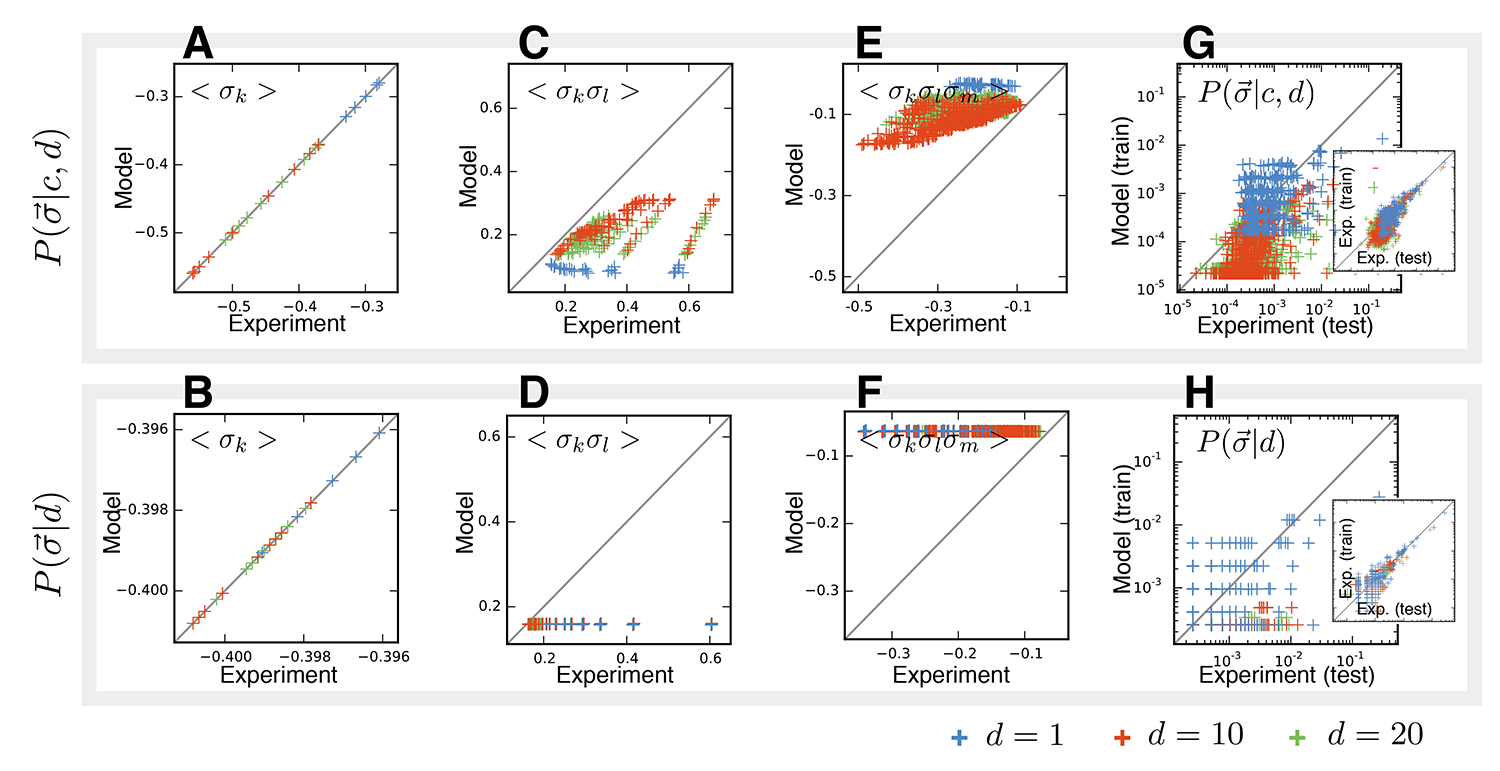

Supplement: S3 Fig — Spin statistics at three different contact distances, d. Top row is for models conditioned on contact, P(σ→|c,d), whereas the bottom row is for models that are regardless of contact, P(σ→|d). (A, B) Site average statistics, 〈σk〉. (C, D) Pairwise correlation statistics, 〈σlσk〉. (E, F) Three body correlation statistics, 〈σkσlσm〉. (G, H) The probability of σ→ from the model versus the observed frequency from contact maps (G) and from the genome (H). For reference the insert compares experimentally measured spin-vector probabilities from training versus testing data. (TIF) [file pcbi.1005956.s004.tif]

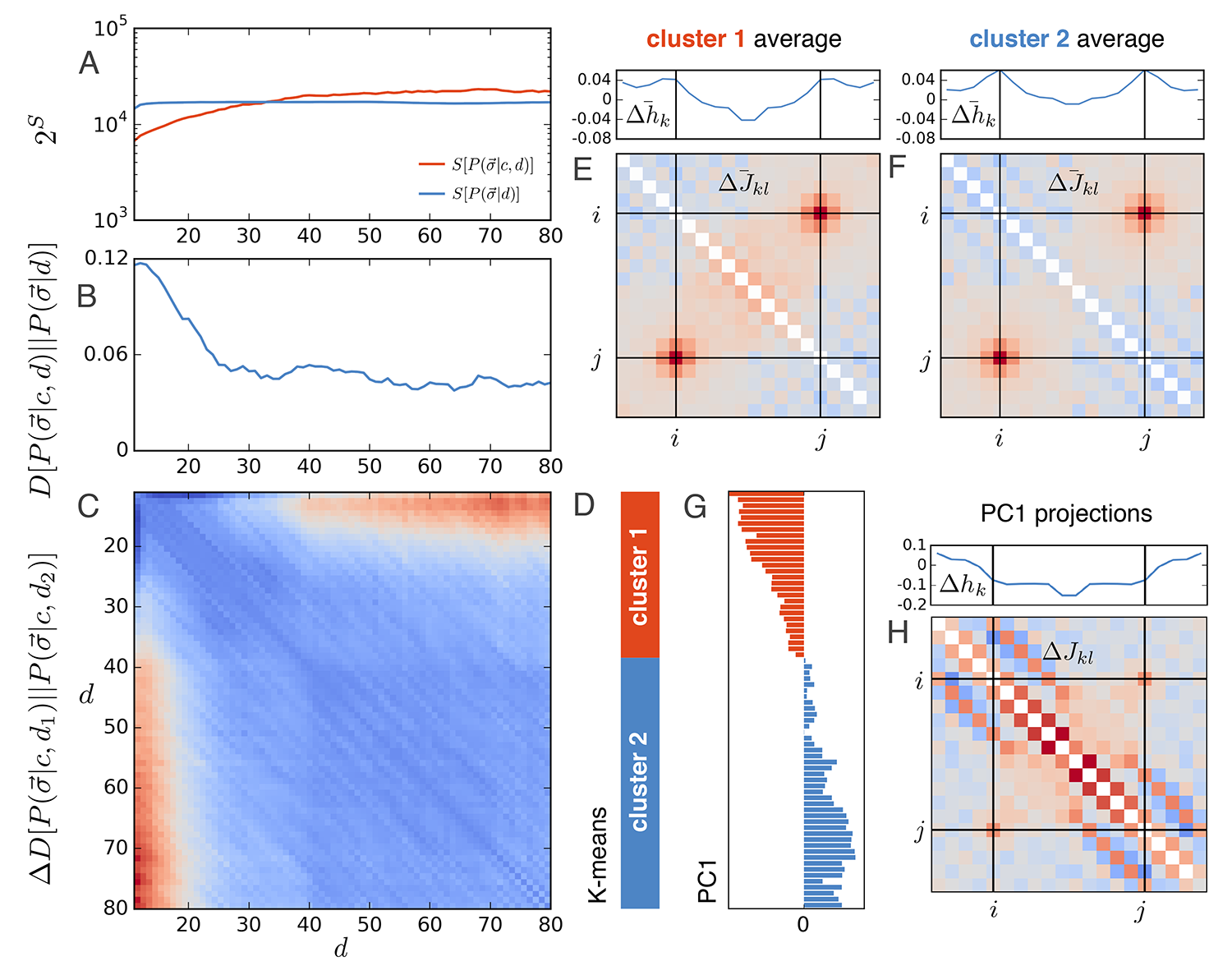

Supplement: S4 Fig — (A) Average number of structures (2S) stored in the probability distributions of neighbors given contact between i and j, P(σ→|c,d), and irrespectively of contact, P(σ→|d), as a function of distance of contact d = |j − i|. (B) Kullback-Liebler divergence between probability distribution of neighbors given contact and irrespectively of contact as a function of distance. (C) Kullback-Liebler divergence between the contacting distributions at different distances of contact minus Kullback-Liebler divergence between the background distributions: ΔD[P(σ→|c,d1)||P(σ→|c,d2)]=D[P(σ→|c,d1)||P(σ→|c,d2)]-D[P(σ→|d1)||P(σ→|d2)]. Blue indicates low values whereas red indicates high values. (D) A vector concatenating the difference between energetic coefficients for neighborhoods in contact and background, Δhkd=hkc,d-hkbg,d and ΔJkld=Jklc,d-Jklbg,d, was built at every distance of contact. K-means clustering of the set of coefficient vectors naturally separated them into two clusters, one for d < 390 Kbp and another for d ≥ 390 Kbp. (E) Average of energetic coefficient vectors in K-means cluster 1. Blue indicates negative values whereas red indicates positive values. (F) Average of energetic coefficient vectors in K-means cluster 2. Blue indicates negative values whereas red indicates positive values. (G) Principal Component Analysis was applied to the set of energetic coefficient vectors. PC1 separates vectors into two two clusters, delimited at 390 Kbp, the same distance that separates K-means clusters. (H) Coefficient vectors projected into PC1 highlight the differences between the coefficients in the two clusters. Blue indicates negative values whereas red indicates positive values. (TIF) [file pcbi.1005956.s005.tif]
